# Validation of the GRade, Age, Nodes and Tumor (GRANT) score within the Surveillance Epidemiology and End Results (SEER) database: A new tool to predict survival in surgically treated renal cell carcinoma patients

**DOI:** 10.1038/s41598-019-49250-6

**Published:** 2019-09-13

**Authors:** Sebastiano Buti, Pierre I. Karakiewicz, Melissa Bersanelli, Umberto Capitanio, Zhe Tian, Alessio Cortellini, Satoru Taguchi, Alberto Briganti, Francesco Montorsi, Francesco Leonardi, Marco Bandini

**Affiliations:** 1 Medical Oncology Unit, University Hospital of Parma, Via Gramsci 14, 43126 Parma, Italy; 2 Centre de recherche du Centre Hospitalier de l’Université de Montréal (CR-CHUM) and Institut du cancer de Montréal, Montréal, Québec Canada; 3 Medicine and Surgery Department, University of Parma, Via Gramsci 14, 43126 Parma, Italy; 4 Division of Oncology/Unit of Urology, URI, IRCCS Ospedale San Raffaele, Vita-Salute San Raffaele University, Milan, Italy; 5 Department of Biotechnological and Applied Clinical Sciences, University of L’Aquila, L’Aquila, Italy; 6 Department of Urology, Kyorin University Faculty of Medicine, Tokyo, Japan

**Keywords:** Nomograms, Urological cancer

## Abstract

The purpose of the present study was to validate the new GRade, Age, Nodes and Tumor (GRANT) score for renal cell carcinoma (RCC) prognostication within a large population of patients. Within the Surveillance, Epidemiology, and End Results database, we identified patients with either clear-cell or papillary RCC, who underwent nephrectomy between 2001 and 2015. Harrell’s C-Index, calibration plot and decision curve analysis were used to validate the GRANT model using a five-risk group stratification (0 vs. 1 vs. 2 vs. 3 vs. 4 risk factors). The primary endpoint was overall survival (OS) at 60 months. The analyses were repeated according to the histologic subgroup. The overall population included 73217 cases; 60900 with clear-cell RCC and 12317 with papillary histology, respectively. According to a five-risk group stratification, 23985 patients (32.8%) had no risk factor (0), 35019 (47.8%) had only one risk factor (1), 13275 (18.1%) had risk score 2854 (1.2%) had 3 risk factors and 84 (0.1%) of cases had a GRANT score of 4, respectively. At 60 months, OS rates as determined by the GRANT score were respectively 94% (score 0) vs. 86% (score 1) vs. 76% (score 2) vs. 46% (score 3) vs. 16% (score 4). In both histologic subtypes, the GRANT score yielded good calibration and high net benefit. OS C-Index values were 0.677 and 0.650 for clear-cell and papillary RCC at 60 months after surgery, respectively. In conclusion, the GRANT score was validated with a five-risk group stratification in a huge population from the SEER database, offering a further demonstration of its reliability for prognostication in RCC.

## Introduction

Renal cell carcinoma (RCC) is responsible for 5% and 3% of all malignancies in men and women, respectively representing the sixth and the tenth most common solid tumor^1^. The main therapeutic approach in early stage RCC is represented by radical or partial nephrectomy, even though a recurrence occurs in approximately 30–40% of patients^2^. The TNM staging system and further prognostic or predictive models, developed to estimate the risk of recurrence and the probability of survival after nephrectomy, are helpful to customize the follow-up schedule, for the patient-tailored counseling and to select individuals more likely to benefit from adjuvant treatments^3–10^.

The best-known nomograms for the risk assessment after surgical treatment of localized RCC are the University of California Los Angeles Integrated Staging System (UISS), predicting overall survival (OS) in patients with RCC regardless of the histologic subtype, and the Mayo Clinic Stage, Size, Grade and Necrosis (SSIGN) model, predicting cancer-specific survival in patients with clear-cell RCC^3–5^.

A plethora of further prognostic models have been reported in the literature^6–10^. Among them, the Karakiewicz nomogram was shown to be superior to other validated models (the Kattan, the Sorbellini and the Leibovich nomograms) predicting survival outcomes in localized RCC^11^. Nevertheless, all these nomograms are currently little used due to their relative complexity of calculation and to the scarce availability of some required parameters. An impediment to the widespread clinical adoption and to the external validation of the SSIGN and the Leibovich scores was represented by their reliance on tumor necrosis, a pathological variable without standardized definition, not quickly available at most centers (especially in the frequent context of the private practice) and with no consensus on the correct reporting method^12^.

An easier nomogram, recently created and validated by our group, is the GRANT score^13^. The name is an acronym of the four risk factors included in the score calculation: Grade (Fuhrman grading of the tumor), Age (patient’s age), Nodes (pathological nodal status, pN) and Tumor (pathological tumor size, pT). The model was developed from an original score initially tested within a phase III randomized trial of adjuvant immunotherapy after radical nephrectomy^14^. It was then validated in the large prospective population of the ASSURE adjuvant trial^15^, using a two-risk group stratification. Its prognostic value in terms of disease-free survival (DFS) and OS^13^ was demonstrated in this setting, as evidenced by the respective accuracy values of 0.589 and 0.613, with a better performance compared to that of the UISS model in the same patient population^15^.

After the validation of the model within patient populations from clinical trials, the aim of the present study was the validation of the GRANT prognostic score system using a five-risk group stratification, in a large North American population-based dataset.

## Patients and Methods

### Data source

The study cohort was identified among individuals diagnosed with RCC (International Classification of Disease for Oncology C64.9) within the Surveillance, Epidemiology, and End Results (SEER) database. The SEER database collects patient demographics and publishes cancer incidence and survival data from several cancer registries. It represents a sample of ~26% from the United States population and approximates the United States overall population in terms of demographic composition, as well as of cancer incidence and mortality. Informed consent and ethical approval were not applicable for the present retrospective, population-based study. All methods were carried out in accordance with relevant guidelines and regulations.

### Study population

Only patients affected by renal parenchymal tumors, aged ≥18 years, and treated with partial or radical nephrectomy between 2001 and 2015 were included from the SEER database. The main inclusion criteria was histologically confirmed (International Classification of Disease for Oncology C64.9^16^) non-metastatic (M0 at diagnosis) RCC stage pT_1-4_N_any_. Death certificate only, autopsy cases, and patients with bilateral tumors were excluded^16,17^. The histological subtypes allowed for the inclusion were clear-cell RCC (ccRCC - histologic code 8310) and papillary RCC (pRCC - histologic code 8260). Further exclusion criteria consisted of unknown tumor grade, lymph node status, T classification, age and follow-up data.

### Variable definition

Data on age, American Joint Committee on Cancer (AJCC 8^th^ edition)-based T and N stages were acquired at the time of diagnosis. Additional variables consisted of race (Black, White, Asian and other), marital status (married, never married, previously married, unknown), gender, year of surgery, socioeconomic status (low vs high), tumor grade (G1, G2, G3 and G4), histologic subtype (clear-cell and papillary) and surgical treatment (radical and partial nephrectomy). Medians and interquartile ranges, as well as frequencies and proportions, were reported for continuous and categorical variables, respectively. The statistical significance of differences in medians and proportions was evaluated with the Kruskal-Wallis and χ^2^ tests.

### Statistical analyses

The aim of the study was the validation of the GRANT score in an independent real-life dataset. The parameters for the calculation of the score were the subsequent: Tumor Grade 1-2 vs. 3-4, Age ≤60 years vs. >60 years, pN_0_-N_X_ vs. pN_1_, pT_1_-T_3a_ vs. all others (any pT_3b_, pT_3c_, pT_4_).

Patients were given one point for each of age >60 years, tumor grade >2, pathologic T-stage of pT_3b_, pT_3c_ or pT_4_, and pathologic N-stage other than N_0_ or N_X_, with a resulting score ranging from 0 to 4^13^.

In the first step of our analyses, we validated the GRANT score using the five-risk group stratification (0 vs. 1 vs. 2 vs. 3 vs. 4 risk factors), within the overall population. In the second step, we validated the GRANT score (using the five-risk group stratification) within the two histologic patient subgroups, respectively clear-cell and papillary RCC. In all analytical steps, the Kaplan-Meier method was used to plot OS rates by risk groups, in order to graphically explore the OS rate differences between the GRANT groups. The validation of the GRANT score was further investigated through the development of a Cox-based model for prediction of 60-month mortality rates after surgery. Such model was internally developed within the SEER population (Online Resource, Supplementary Table 1). Subsequently, the performance of the model was assessed in terms of discrimination, using the Harrell’s c-index^18^. Furthermore, calibration plots^19^ and decision curve analysis (DCA)^20^ were generated based on predicted probabilities derived from the use of the GRANT score, with internal development. Calibration was assessed in terms of departures from ideal agreement between predicted and observed probabilities. Similarly, the net benefit derived from the use of the GRANT score within the SEER population was described with the DCA analysis in terms of net benefit (Y axis) from the “treat-all” and “treat-none” option. All the statistical tests were two-sided with a level of significance set at *P* < 0.05. The analyses were performed using the R software environment for statistical computing and graphics (version 3.3.0).

### Research involving Human Participants and/or Animals

The procedures followed for the present Original Research were in accordance with the declaration of Helsinki.

## Results

### Study population

**Figure 1:**
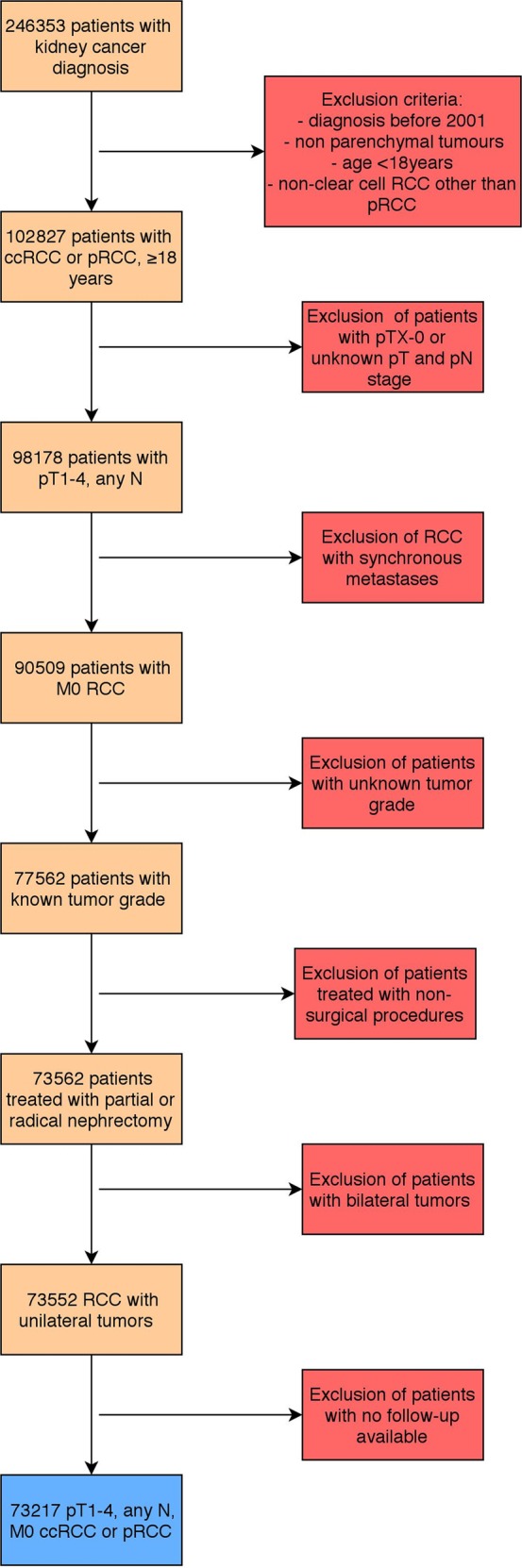
Diagram flowchart of inclusion and exclusion criteria of the study. RCC = renal cell carcinoma; ccRCC = clear-cell renal cell carcinoma; pRCC = papillary renal cell carcinoma

The selection process resulted in 73217 patients included in the analysis (Fig. 1). Of them, 23985 (32.8%), 35019 (47.8%), 13275 (18.1%), 854 (1.2%) and 84 (0.1%) patients had a GRANT score of 0, 1, 2, 3 and 4, respectively. The characteristics of patients were listed in Table 1.

**Table 1:** Clinical and pathological characteristics of 73217 pT_1-4_N_any_ surgically treated renal cell carcinoma patients.

Variable	Category/Status	Overall (*N* = 73217)	Clear-Cell (*N* = 60900, 83.2%)	Papillary (*N* = 12317, 16.8%)	*P* values
Age at diagnosis (years)	Range	53–70	52–70	54–70	<0.0001
Gender	Female	26,464 (36.1)	23,563 (38.7)	2,901 (23.6)	<0.0001
	Male	46,753 (63.9)	37,337 (61.3)	9,416 (76.4)	
Race	Other	1,130 (1.5)	1,021 (1.7)	109 (0.9)	<0.0001
	White	60,477 (82.6)	52,008 (85.4)	8,469 (68.8)	
	Asian	3,894 (5.3)	3,553 (5.8)	341 (2.8)	
	Black	7,716 (10.5)	4,318 (7.1)	3,398 (27.6)	
Marital status	Married	46,395 (63.4)	38,814 (63.7)	7,581 (61.5)	<0.0001
	Never Married	10,378 (14.2)	8,351 (13.7)	2,027 (16.5)	
	Previously Married	13,121 (17.9)	11,002 (18.1)	2,119 (17.2)	
	Unknown	3,323 (4.5)	2,733 (4.5)	590 (4.8)	
Socioeconomic status	high	36,086 (49.3)	30,170 (49.5)	5,916 (48)	0.002
	low	37,128 (50.7)	30,727 (50.5)	6,401 (52)	
Histology	Clear-cell	60,900 (83.2)	60,900 (100)	0 (0)	<0.0001
	Papillary	12,317 (16.8)	0 (0)	12,317 (100)	
Tumor Grade	G1/G2	50,116 (68.4)	42,060 (69.1)	8,056 (65.4)	<0.0001
	G3/G4	23,101 (31.6)	18,840 (30.9)	4,261 (34.6)	
T Stage	T1-2-3a	71,819 (98.1)	59,621 (97.9)	12,198 (99)	<0.0001
	T3b-3c-4	1,398 (1.9)	1,279 (2.1)	119 (1)	
N stage	0-X	72,139 (98.5)	60,150 (98.8)	11,989 (97.3)	<0.0001
	1	1,078 (1.5)	750 (1.2)	328 (2.7)	
Surgical approach	Partial nephrectomy	25,115 (34.3)	19,622 (32.2)	5,493 (44.6)	<0.0001
	Radical nephrectomy	48,102 (65.7)	41,278 (67.8)	6,824 (55.4)	
GRANT score	0	23,985 (32.8)	20,424 (33.5)	3,561 (28.9)	<0.0001
	1	35,019 (47.8)	28,985 (47.6)	6,034 (49)	
	2	13,275 (18.1)	10,726 (17.6)	2,549 (20.7)	
	3	854 (1.2)	703 (1.2)	151 (1.2)	
	4	84 (0.1)	62 (0.1)	22 (0.2)	

### GRANT score validation within the overall cohort

**Figure 2:**
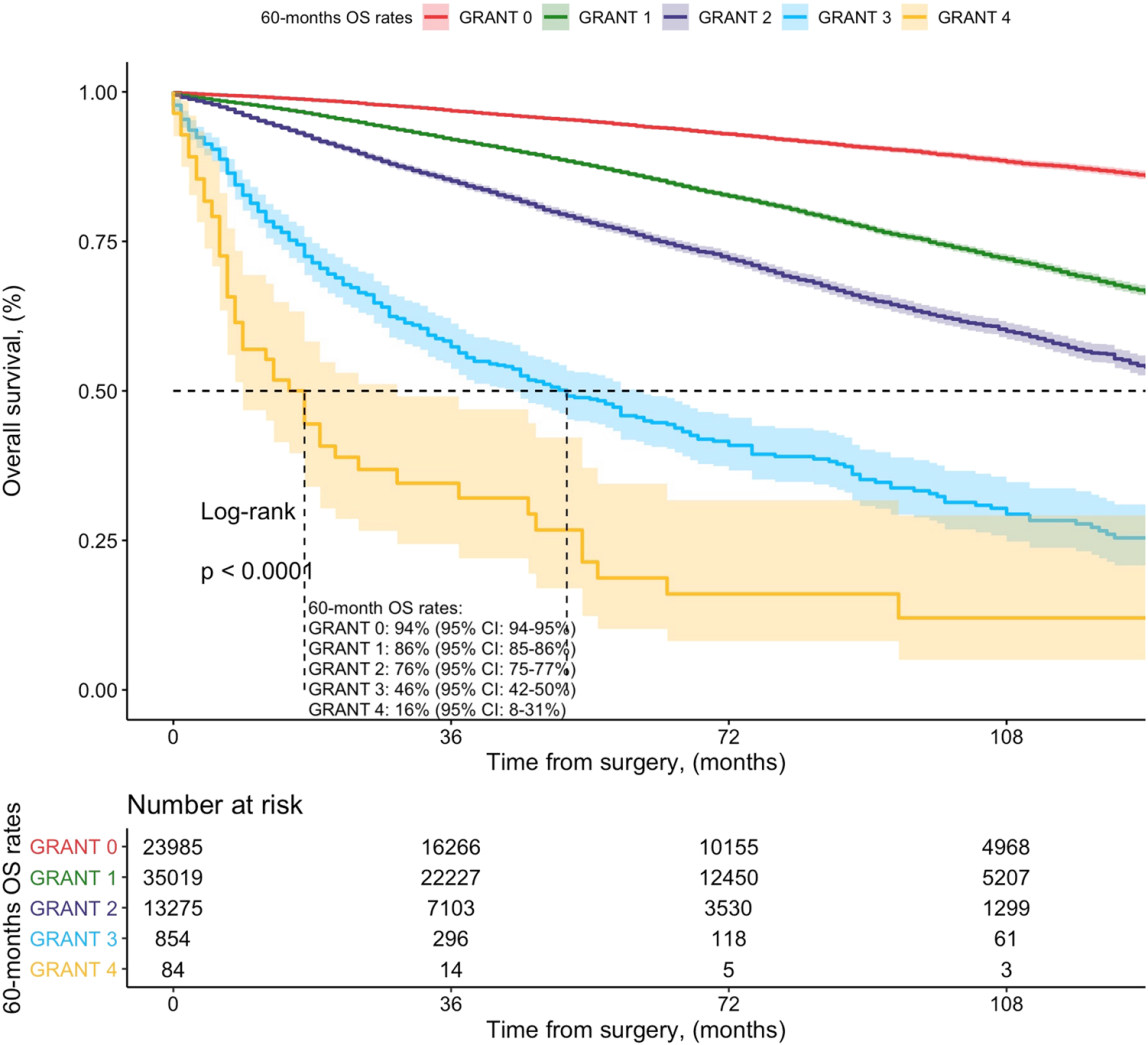
Kaplan-Meier plots exploring overall survival (OS) rates of 73217 patients treated with radical or partial nephrectomy within the SEER registry, between 2001 and 2015. Patients were stratified according to the GRANT score: GRANT score 0 (23985, 32.8%), GRANT score 1 (35019, 47.8%), GRANT score 2 (13275, 18.1%), GRANT score 3 (854, 1.2%), GRANT score 4 (84, 0.1%). Median follow-up was 60, 52, 39, 21 and 8 months for GRANT score group 0, 1, 2, 3 and 4, respectively.

**Figure 3:**
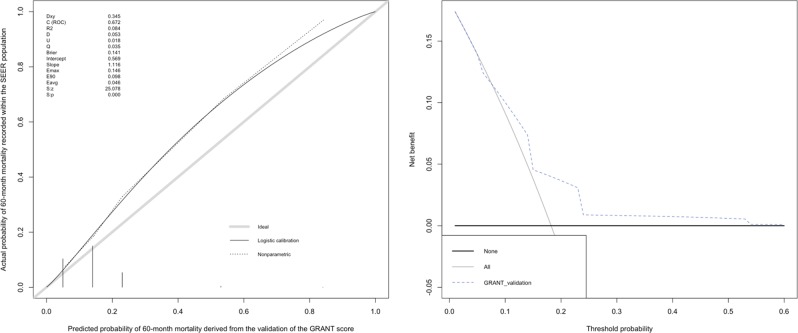
C-Index, calibration plot and decision curve analysis of the GRANT score (0 vs. 1 vs. 2 vs. 3 vs. 4) within the overall (59004) SEER population predicting OS at 60 months.

At 60 months, OS rates by 5 risk groups as determined by the GRANT score were 94% (score 0) vs. 86% (score 1) vs. 76% (score 2) vs. 46% (score 3) vs. 16% (score 4), respectively (Fig. 2). OS C-Index value was 0.672 at 60 months. According to the calibration plot, we had small departures from ideal agreement between predicted and observed probabilities. Here, the GRANT score appeared to slightly underpredict the risk of mortality at 60 months compared to the actual 60-month mortality rates that we have registered within the SEER population. DCA illustrated the net benefit of the model, which was higher than the “treat-all” and “treat-none” options at threshold probabilities above 10% and below 50% (Fig. 3).

### GRANT score validation within clear-cell and papillary patient sub-cohorts

Overall, 60900 and 12317 patients had clear-cell and papillary histology, respectively. Among clear-cell RCC cases, 20424 (33.5%), 28985 (47.6%), 10726 (17.6%), 703 (1.2%) and 62 (0.1%) patients respectively had a GRANT score of 0, 1, 2, 3 and 4. Otherwise, 3561 (28.9%), 6034 (49%), 2549 (20.7%), 151 (1.2%) and 22 (0.2%) papillary RCC patients had a GRANT score of 0, 1, 2, 3 and 4, respectively.

Stratified OS rates by risk score within the histologic subgroups are shown in Supplementary Fig. 1 (Online Resource). OS C-Index values were respectively 0.677 and 0.650 for clear-cell and papillary RCC at 60 months after surgery. Calibration plots and DCA for clear-cell and papillary RCC patients were showed in Supplementary Fig. 2 and Supplementary Fig. 3 (Online Resource).

## Discussion

In the development of predictive algorithms, there is often a compromise between predictive accuracy and easiness of use. Whereas adding more variables and data can increase the accuracy of a prognostic model, beyond a certain limit it also increases the complexity, without significantly improving the predictive ability of the tool^11^. The everyday employment of validated nomograms in clinical practice is unusual, maybe due to the complexity in the score calculation and to the possible lack of the required data. The GRANT score, especially when compared to other models, is easy to calculate. The four required parameters are almost always available for nephrectomized patients, including a simple demographic element (age) and common histopathological data.

The major strength of the present study is represented by the huge validation sample, with the full representation of all risk categories. Contrariwise to the original validation setting, given by a trial of adjuvant therapy^13^, the present population is inclusive of low risk patients (e.g. T1, N0, G1 tumors). This element can explain the even better performance of the GRANT score with a five-risk group stratification (C-Index 0.672 at 60 months), beyond its original version with the two-risk group stratification^13^. The current validation of this alternative use of the score provides a reliable and useful prognostication tool for unselected surgical patient populations.

A further improvement, compared to the previous validation setting, is represented herein by the use of the TNM 8^th^ edition. Indeed, the TNM classification has been modified overtime: in the 6^th^ TNM edition (2002), pT3a was defined as the extension to ‘perinephric tissue, renal sinus or contiguous into adrenal gland’ (renal vein involvement implied T3b), while in the 7^th^ TNM (2010) it included ‘perinephric tissue, renal sinus or renal vein’ (the adrenal gland involvement was defined as T4)^2,21^. Additionally, in the 8^th^ TNM system, the invasion of pelvic-caliceal system was included among T3a. Similarly, pN classification was changed overtime. In the last two editions of the TNM, pN2 was eliminated and patients with regional nodal metastases were identified as N1, regardless of their number or site^22^. This update actualizes the GRANT score for the current clinical use in a real-life setting.

Moreover, the long follow-up of this patient population allowed to show that the curves of each risk group maintain separation over the fifth year after surgery, representing a not negligible advantage of the model, considering that at least 22% of the relapsing patients have their recurrence more than 5 years after nephrectomy^23^.

The present work finally offers a usable clinical tool for a relevant subgroup of non-clear cell RCC, since the GRANT model is now one of the few specifically validated in a wide papillary RCC population. Indeed, it shows valuable discrimination and good calibration also within this subgroup (0.650 C-Index), with comparable results to those of the clear-cell patient subgroup.

A consideration is due about the only clinical factor that distinguish the GRANT score from the majority of the other nomograms. The “age” parameter, apparently abused for cancer patient selection and counseling in the everyday clinical practice, is inexplicably trapped in the field of prognostication. Despite age may obviously represent an independent prognostic element, even for healthy individuals (the greater the age, the greater the risk of dying), it has been previously included within only one prognostic model^24^. Its inclusion could be crucially timely, especially in the field of the new anticancer immunotherapy, on a hand considering the potentially negative prognostic weight of an older age, on the other taking into account the usability of immune checkpoint inhibitors in the elderly, with the opportunity to prolong survival irrespective of age^25^.

The limitations of the present study are represented by the retrospective nature, the relatively low C-Index (despite it was comparable to those of previous similar models by the literature^4–10^), the sub-optimal extent of the median follow-up and the lack of data in terms of DFS.

The choice of validating the GRANT score with a five-risk group stratification was due both to the aim to test the score in a real-world population (including low risk patients) and to the expected potentially significant prognostic impact of the number of single risk factors used for allocating patients in cumulative-risk groups, similar to what happens in the case of other prognostic models for metastatic RCC^26^. On the other hand, it is known that the prognostic weight of each single factor included in a model is largely unreliable^24^. The reliability of a score for a true prognostication is based on the concept that the final effect of all the parameters is greater than the sum of the single factors’ weight.

## Conclusions

The GRANT score was successfully validated with a five-risk group stratification in a huge RCC patient population from the SEER database. This successful application of the GRANT model represents an additional demonstration of its validity, combining easiness of calculation and reliability of prognostication for surgically treated RCC patients.

## Supplementary information

Supplementary Materials
