# Author Correction: Validation of the GRade, Age, Nodes and Tumor (GRANT) score within the Surveillance Epidemiology and End Results (SEER) database: A new tool to predict survival in surgically treated renal cell carcinoma patients

**DOI:** 10.1038/s41598-020-59358-9

**Published:** 2020-02-05

**Authors:** Sebastiano Buti, Pierre I. Karakiewicz, Melissa Bersanelli, Umberto Capitanio, Zhe Tian, Alessio Cortellini, Satoru Taguchi, Alberto Briganti, Francesco Montorsi, Francesco Leonardi, Marco Bandini

**Affiliations:** 1 Medical Oncology Unit, University Hospital of Parma, Via Gramsci 14, 43126 Parma, Italy; 2 Centre de recherche du Centre Hospitalier de l’Université de Montréal (CR-CHUM) and Institut du cancer de Montréal, Montréal, Québec Canada; 3 Medicine and Surgery Department, University of Parma, Via Gramsci 14, 43126 Parma, Italy; 4 Division of Oncology/Unit of Urology, URI, IRCCS Ospedale San Raffaele, Vita-Salute San Raffaele University, Milan, Italy; 5 Department of Biotechnological and Applied Clinical Sciences, University of L’Aquila, L’Aquila, Italy; 6 Department of Urology, Kyorin University Faculty of Medicine, Tokyo, Japan

Correction to: *Scientific Reports* 10.1038/s41598-019-49250-6, published online 13 September 2019

The original version of this Article contained a typographical error in the Abstract.

“According to a five-risk group stratification, 23985 patients (32.8%) had no risk factor (0), 35019 (47.8%) had only one risk factor (1), 13275 (18.1%) had risk score 2854 (1.2%) had 3 risk factors and 84 (0.1%) of cases had a GRANT score of 4, respectively.”

now reads:

“According to a five-risk group stratification, 23985 patients (32.8%) had no risk factor (0), 35019 (47.8%) had only one risk factor (1), 13275 (18.1%) had risk score 2, 854 (1.2%) had 3 risk factors and 84 (0.1%) of cases had a GRANT score of 4, respectively.”

This has now been corrected in the PDF and HTML versions of the Article.

