# Supplementary material for: Validation of the GRade, Age, Nodes and Tumor (GRANT) score within the Surveillance Epidemiology and End Results (SEER) database: A new tool to predict survival in surgically treated renal cell carcinoma patients
Source: Sci Rep. 2019 Sep 13;9:13218. doi: 10.1038/s41598-019-49250-6 (PMC6744465; doi:10.1038/s41598-019-49250-6)
Supplement: Supplementary file 1 — Supplementary Materials [file 41598_2019_49250_MOESM1_ESM.pdf]

Validation of the GRade, Age, Nodes and Tumor (GRANT) score within the Surveillance Epidemiology and End Results (SEER) database: easy-to use overall survival prediction in surgically treated renal cell carcinoma patients

Sebastiano Buti[1], Pierre I. Karakiewicz[2], Melissa Bersanelli[1,3§], Umberto Capitanio[4], Zhe Tian[2], Alessio Cortellini[5], Satoru Taguchi[6], Alberto Briganti[4], Francesco Montorsi[4], Francesco Leonardi[1] and Marco Bandini[4].

[1] Medical Oncology Unit, University Hospital of Parma, Via Gramsci 14, 43126, Parma, Italy

[2] Centre de recherche du Centre Hospitalier de l'Université de Montréal (CR-CHUM) and Institut du cancer de Montréal, Montréal, Québec, Canada

[3] Medicine and Surgery Department, University of Parma, Via Gramsci 14, 43126, Parma, Italy

[4] Division of Oncology/Unit of Urology, URI, IRCCS Ospedale San Raffaele, Vita-Salute San Raffaele University, Milan, Italy

[5] Department of Biotechnological and Applied Clinical Sciences, University of L'Aquila, L'Aquila, Italy.

[6] Department of Urology, Kyorin University Faculty of Medicine, Tokyo, Japan

[§]**Corresponding Author:** Dr Melissa Bersanelli, Medical Oncology Unit, University Hospital of Parma, Via Gramsci 14, 43126, Italy, phone number: +39 0521 702316, e-mail address: bersamel@libero.it, ORCID ID: 0000-0002-6527-6281

Supplementary Material

Supplementary Figure 1 - Kaplan-Meier plots of overall survival according to histology groups. A. Kaplan-Meier plots of overall survival (OS) in 60,900 clear-cell RCC patients treated with radical or partial nephrectomy between 2001 and 2015. Here, 20424 (33.5%), 28985 (47.6%), 10726 (17.6%), 703 (1.2%) and 62 (0.1%) clear-cell RCC patients present GRANT score 0, 1, 2, 3 and 4, respectively. Median follow-up was 60, 52, 39, 21 and 8 months in the 0, 1, 2, 3 and 4 risk groups, respectively. **B.** Kaplan-Meier plots of OS in 12,317 papillary RCC patients treated with radical or partial nephrectomy between 2001 and 2015. Here, 23561 (28.9%), 6034 (49%), 2549 (20.7%), 151 (1.2%) and 22 (0.2%) papillary RCC patients present GRANT score 0, 1, 2, 3 and 4, respectively. Median follow-up was 61, 52, 41, 21 and 9 months in the 0, 1, 2, 3 and 4 risk groups, respectively.

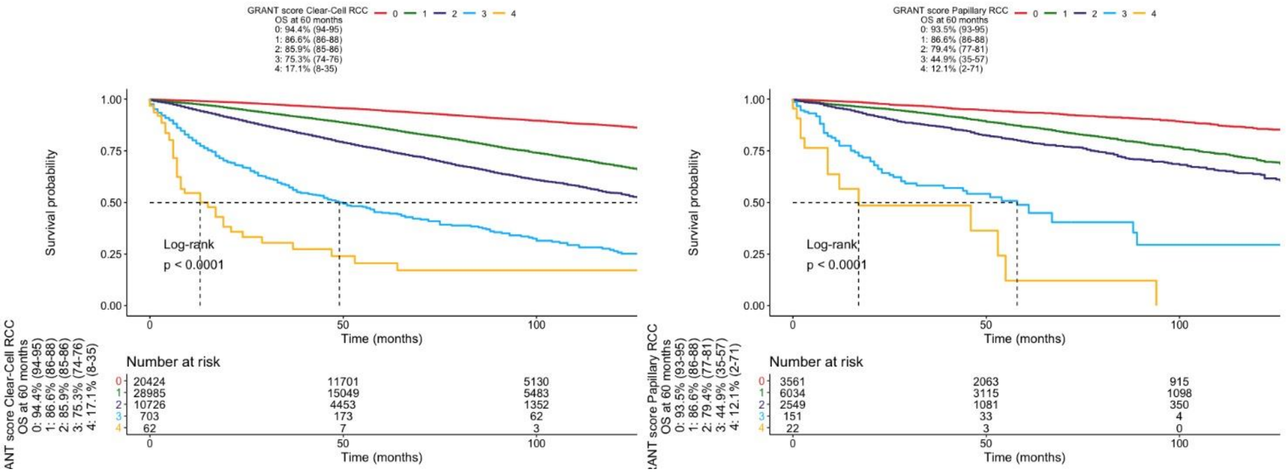

Supplementary Figure 2: C-Index, calibration plot and decision curve analysis of the GRANT score (0 vs. 1 vs. 2 vs. 3 vs. 4) within the clear-cell RCC (60,900) population predicting OS at 60 months.

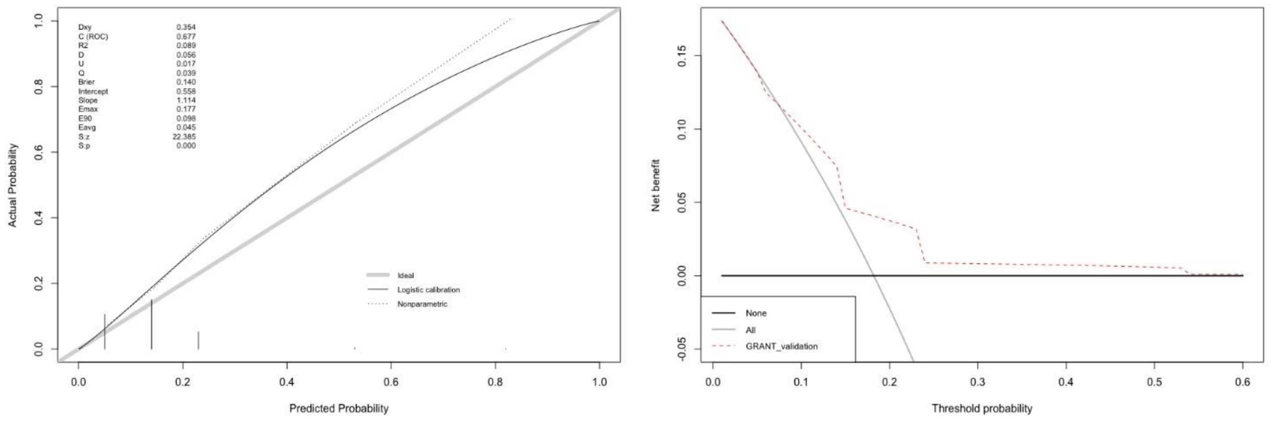

Supplementary Figure 3: C-Index, calibration plot and decision curve analysis of the GRANT score (0 vs. 1 vs. 2 vs. 3 vs. 4) within the papillary RCC (12,317) population predicting OS at 60 months.

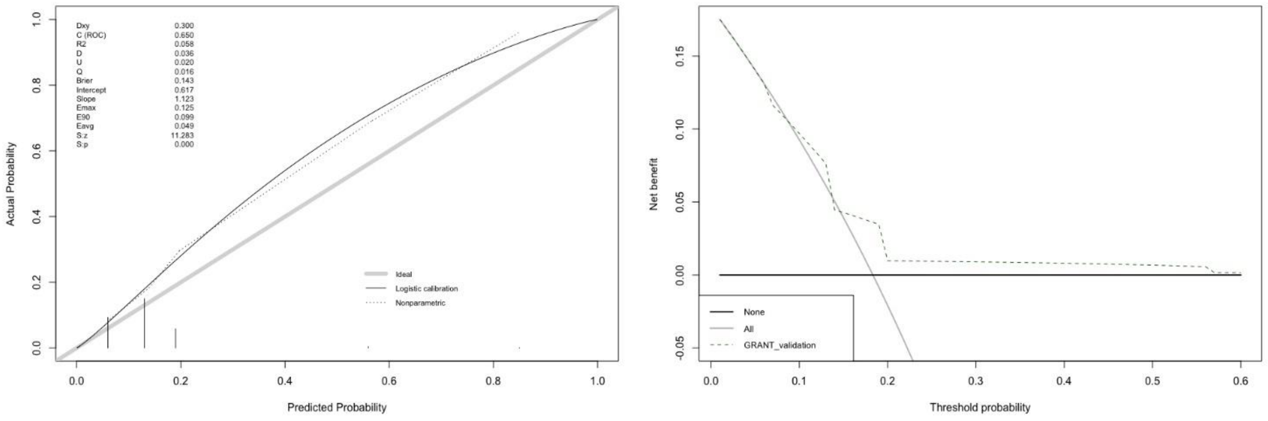

Supplementary Table 1 - Cox regression models and Hazard Ratios values according to different patient sub-groups.

Cox regression models *	HR	95% CI	p Values
GRANT score 1 (N= 35019) vs. GRANT score 0 (N= 23985)	2.50	2.36-2.63	<0.0001
GRANT score 2 (N= 13275) vs. GRANT score 0 (N= 23985)	4.04	3.80-4.29	<0.0001
GRANT score 3 (N= 854) vs. GRANT score 0 (N= 23985)	10.41	9.34-11-61	<0.0001
GRANT score 4 (N= 84) vs. GRANT score 0 (N= 23985)	24.02	18.24-31-62	<0.0001
GRANT score 2 (N= 13275) vs. GRANT score 1 (N= 35019)	1.62	1.55-1.69	<0.0001
GRANT score 3 (N= 854) vs. GRANT score 1 (N= 35019)	4.17	3.77-4.62	<0.0001
GRANT score 4 (N= 84) vs. GRANT score 1 (N= 35019)	9.62	7.33-12.63	<0.0001
GRANT score 3 (N= 854) vs. GRANT score 2 (N= 13275)	2.58	2.32-2.86	<0.0001
GRANT score 4 (N= 84) vs. GRANT score 2 (N= 13275)	5.95	4.53-7.82	<0.0001
GRANT score 4 (N= 84) vs. GRANT score 3 (N= 854)	2.31	1.73-3.07	<0.0001
